# The histone methyltransferase inhibitor A-366 enhances hemoglobin expression in erythroleukemia cells upon co‐exposure with chemical inducers in culture

**DOI:** 10.1186/s40709-020-00132-3

**Published:** 2021-01-06

**Authors:** Christos I. Papagiannopoulos, Nikoleta F. Theodoroula, Konstantinos A. Kyritsis, Melpomeni G. Akrivou, Maria Kosmidou, Konstantina Tsouderou, Nikolaos Grigoriadis, Ioannis S. Vizirianakis

**Affiliations:** 1 Laboratory of Pharmacology, School of Pharmacy, Aristotle University of Thessaloniki, 54124 Thessaloniki, Greece; 2 FunPATH (Functional Proteomics and Systems Biology Research Group at AUTH) Research Group, Balkan Center, KEDEK-Aristotle University of Thessaloniki, 57001 Thessaloniki, Greece; 3 Biogenea Pharmaceuticals Ltd, Thessaloniki, Greece; 4 Department of Life and Health Sciences, University of Nicosia, 1700 Nicosia, Cyprus

**Keywords:** Methyltransferases, Erythroleukemia cells, Differentiation, A-366, Chemical inducers

## Abstract

**Background:**

Erythroleukemia is caused by the uncontrolled multiplication of immature erythroid progenitor cells which fail to differentiate into erythrocytes. By directly targeting this class of malignant cells, the induction of terminal erythroid differentiation represents a vital therapeutic strategy for this disease. Erythroid differentiation involves the execution of a well-orchestrated gene expression program in which epigenetic enzymes play critical roles. In order to identify novel epigenetic mediators of differentiation, this study explores the effects of multiple, highly specific, epigenetic enzyme inhibitors, in murine and human erythroleukemia cell lines.

**Results:**

We used a group of compounds designed to uniquely target the following epigenetic enzymes: G9a/GLP, EZH1/2, SMYD2, PRMT3, WDR5, SETD7, SUV420H1 and DOT1L. The majority of the probes had a negative impact on both cell proliferation and differentiation. On the contrary, one of the compounds, A-366, demonstrated the opposite effect by promoting erythroid differentiation of both cell models. A-366 is a selective inhibitor of the G9a methyltransferase and the chromatin reader Spindlin1. Investigation of the molecular mechanism of action revealed that A-366 forced cells to exit from the cell cycle, a fact that favored erythroid differentiation. Further analysis led to the identification of a group of genes that mediate the A-366 effects and include CDK2, CDK4 and CDK6.

**Conclusions:**

A-366, a selective inhibitor of G9a and Spindlin1, demonstrates a compelling role in the erythroid maturation process by promoting differentiation, a fact that is highly beneficial for patients suffering from erythroleukemia. In conclusion, this data calls for further investigation towards the delivery of epigenetic drugs and especially A-366 in hematopoietic disorders.

## Background

Erythroleukemia, a subtype of acute myeloid leukemia, is caused by the neoplastic proliferation of myeloid and erythroid progenitor cells that fail to differentiate into erythrocytes [1]. One promising therapeutic strategy for this disease is the induction of terminal erythroid differentiation, which irreversibly changes the phenotype of cancer cells by transforming them into non-dividing erythrocyte-like cells. Erythroid differentiation is executed under a strictly regulated program of gene expression that is guided by cell-fate transcription factors and epigenetic enzymes such as chromatin remodeling and histone-methyltransferases. Epigenetic enzymes induce chemical changes into the genome, or the transcriptome, in the form of methylation or acetylation and drive differentiation progression [2]. Prominent examples of such regulation in erythropoiesis, include DNA methylation at a CpG island upstream of the alpha- and beta-globin genes [3], as well as the Histone 3 lysine 27 trimethylation (H3K27me3), Histone 3 lysine 4 mono-methylation (H3K4me) [4] and Histone 3 lysine 4 di-methylation (H3K4me2) [4–6] marks, which regulate large cohorts of genes.

Epigenetic enzymes represent highly interesting targets in drug development, especially in diseases associated with a modified epigenome [7–11]. Until now, two DNA methyltransferase 1 inhibitors, 5-azacytidine and 5-aza-2΄-deoxycytidine, have received FDA approval for the treatment of various types of cancer as well as the myelodysplastic syndrome [12, 13]. Moreover, vorinostat, a histone deacetylase inhibitor, has also received approval for the treatment of T-lymphoma in patients with progressive, persistent or recurrent form of the disease [14]. Despite the early development, already by 2006, and the success of these drugs in the clinical practice, there is no novel epigenome-modifying treatment that has received approval since then. Drug discovery in the field has been obstructed by the limited understanding of the outcomes of epigenetic alterations as well as the scarcity of the available tools (such as antibodies or selective inhibitors) to thoroughly characterize epigenetic enzymes [15]. To address these issues, research groups and private partners have established consortia such as the Structural Genomics Consortium (SGC), with the aim to solve crystal structures of key epigenetic enzymes and develop highly selective chemical probes. Up to date, the consortium has developed selective inhibitors for key epigenetic enzymes such as EZH2, G9a/GLP, DOT1L, PRMT3, BET, SETD8 and DOT1L. Each small-molecule inhibitor and antagonist was developed and extensively tested in order to demonstrate potent cellular activity and selective inhibition.

Previously published data from our group have shown that hypomethylating agents possess the capacity to affect the erythroid differentiation program of established cellular erythroleukemia models (murine erythroleukemia cells—MEL and K562) [1, 16–23]. In this work, we report the biological activities of 12 small-molecule protein methyltransferase inhibitors designed to target the following epigenetic enzymes: G9a/GLP, EZH1/2, SMYD2, PRMT3, WDR5, SETD7, SUV420H1 and DOT1L. Interestingly, all but one (A-366 targeting G9a) compounds exert medium to high cytotoxicity, as well as blockade of erythroid differentiation in our cellular models. On the contrary, A-366 appears to facilitate the erythroid maturation of MEL cells, as evident by three experimental assays (western blot, real-time PCR, and cytochemistry). The promising properties of A-366 appear to be dependent on the modulation of critical enzymes and proteins involved in cell cycle progression.

## Methods

### Synthesis and storage conditions of methyltransferase inhibitors

All the compounds used in this study were provided by the Structural Genomics Consortium (SGC), University of Toronto, and they have been delivered to our research group at the Laboratory of Pharmacology, School of Pharmacy, Aristotle University of Thessaloniki, Greece. Moreover, the negative chemical probe for A-366, SGC2097, was also provided by the SGC in order to assess the off-target effects of A-366, since the congener substance SGC2097 lacks the target-inhibiting capacity. All compounds were dissolved in the appropriate DMSO volume to prepare the original 0.1 M concentration stock solution and stored at − 20 °C, in a dark place to avoid photosensitivity issues that may appear over time.

### Cell cultures

The already established permanent cancer model MEL-745 (murine erythroleukemia FLC clone 745) along with human cancer model K562 were handled in a way to maintain their passages with high inducibility erythroid differentiation rates, and used in a routine manner from our research group (Laboratory of Pharmacology, School of Pharmacy, Aristotle University of Thessaloniki, Greece). The murine erythroleukemia MEL-745 cells were obtained from Dr. C. Friend (Division of Cytology, The Sloan-Kettering Institute for Cancer Research, New York, NY, USA). Both cell lines were grown in an incubator under standard conditions.

### Cell propagation and cytotoxicity assessment

The K562 and MEL-745 cell lines were seeded in 24-well plates at an initial concentration of 1 × 10^5^ cells ml^− 1^. The specified concentrations of the compounds used in the cultures were between 1 × 10^− 7^ and 1 × 10^− 4^ M. Taking into account the well-known cytotoxic and possible differentiating action of DMSO, the experiments were designed so that the final concentration of DMSO did not exceed 0.2% v/v of the total culture volume. To estimate the IC_50_ of each compound for each cell line, cells were allowed to grow for 48 h in the presence of the 12 compounds before the measurement of cell proliferation. The cells were counted (cell density; number of cells ml^− 1^) with the aid of the optical microscope (Neubauer counting chamber, Paul Marienfeld GmbH & Co.KG, Lauda-Königshofen, Germany). Cell growth is expressed as a percentage relative to that for the untreated control culture. Subsequently, the calculation of the half-maximal inhibitory concentration (IC_50_) values of each compound for a specific cell line was estimated. Moreover, cell death within cell cultures was also determined using the Trypan blue dye-exclusion method, as previously described [16, 21, 24].

### Assessment of MEL cell differentiation

Parental MEL cells were maintained in Dulbecco’s modified Eagle’s medium as previously described [20, 22, 23]. MEL cell cultures were exposed to HMBA (5 mM), as indicated under the individual figures and the 12 compounds in their IC_50_ values. Following 72 and 96 h of exposure, the accumulation of differentiated (hemoglobin-producing; Bz^+^ cells) cells was assessed cytochemically with benzidine-H_2_O_2_ solution, as previously described [20, 21].

### Cell cycle analysis by flow cytometry

Cell cycle analysis was performed using flow cytometry by measuring the cellular DNA content after PI staining. MEL cells were plated at 6-well plates (5 × 10^5^ cells per well). The cells were then synchronized after serum deprivation for 24 h and were subsequently incubated with the combination formulations for 24, 48, and 72 h. After the specified time points, cells were harvested and fixed in 70% ethanol overnight at 4 °C. The DNA content was measured using the CyStain PI absolute T kit (Partec, Münster, Germany) according to the manufacturer’s guidelines. Analysis was performed using CyFlow Cube 8 (Partec, Münster, Germany), and 40,000 events were recorded. Cell cycle distribution was analyzed using the FCS Express 4 (De Novo software, Los Angeles, CA).

### RNA extraction and reverse transcription‐quantitative polymerase chain reaction (RT-qPCR) analysis

K562 cells were plated with the selected concentration of the three compounds. After treatment for 24 h, the cells were harvested, and total RNA was isolated using the Nucleospin RNA/protein kit (Macherey-Nagel, Germany). After isolation, RNA was tested qualitatively and quantitatively, respectively, by gel electrophoresis assay and by the means of NanoDrop 2000 (Thermo Fisher Scientific, Waltham, Massachusetts, USA) spectrophotometer. RNA samples were subjected to reverse transcription using the QuantiTect Reverse Transcription kit (Qiagen Inc., Chatsworth, CA, USA), as per manufacturer’s instructions. Quantitative RT-PCR analysis was performed using the KAPA SYBR FAST qPCR Kit (KK4602, Kapa Biosystems, Wilmington, MA, USA).

### Western blot analysis

Approximately 10^6^ cells were harvested, by centrifugation at 300×*g* for 5 min, and processed for total protein isolation and western blot analysis. Pelleted cells were washed twice with PBS and lysed in buffer A (10 mM Tris-Cl pH 8.0, 150 mM NaCl, 2% SDS). The lysate appeared highly viscous due to DNA release and, therefore, it was sonicated for 30 s. The samples were then processed for protein quantitation using the BCA assay kit. One volume of protein sample was, consequently, mixed with equal volume of laemmli sample buffer (4% SDS, 20% glycerol, 10% 2-mercaptoethanol, 0.004% bromophenol blue and 0.125 M Tris HCl, pH 6.8) and boiled for 5 min at 95 °C. Twenty µg of total protein material was then loaded on a denaturing 12% SDS-PAGE gel and run until separation. Subsequently, proteins were transferred to a PVDF membrane and blotted with primary antibodies overnight at 4 °C in a shaker and with secondary antibody for 1 h at room temperature. The antibodies used were from Santa Cruz Biotechnology (Finnell Street Dallas, Texas 75220, USA): Hemoglobin β/γ/δ/ε (sc-390668), β-actin (sc-47778), α- tubulin (sc-51503) and m-IgGκBP-HRP (sc-516102).

### Bioinformatic analysis of the Spindlin1-target genes

The Spindlin1-target genes were retrieved from the ChIP-Atlas database [25] using the following parameters; species: *Homo sapiens*, Distance from Transcription Start Site (TSS): ± 10 k. The analysis returned 58 target genes analyzed from ChIP-Seq data in human cell line T778. Functional annotation of the target genes was conducted using the DAVID bioinformatic tool [26]. Network analysis was accomplished with the string database tool [27–30]. Network edges represent protein-protein interactions, sourced from Text mining, Experiments, Databases, Co‑expression, Neighborhood, Gene Fusion and Co‑occurrence. Minimum required interaction score was set to medium confidence 0.400. MCL clustering was performed with an MCL inflation parameter equal to 3.

### Statistical analysis

All experiments were carried out in at least three biological repetitions. Statistical analysis was performed with the GraphPad Prism 6 software (Northside Dr. Suite 560, San Diego, USA), whereas the test used as well as the calculated *p*-values are shown in the legend under each individual figure (*p* < 0.05 was considered to indicate a statistically significant difference).

## Results

### The use of in vitro cell models to study erythropoiesis

**Fig. 1:**
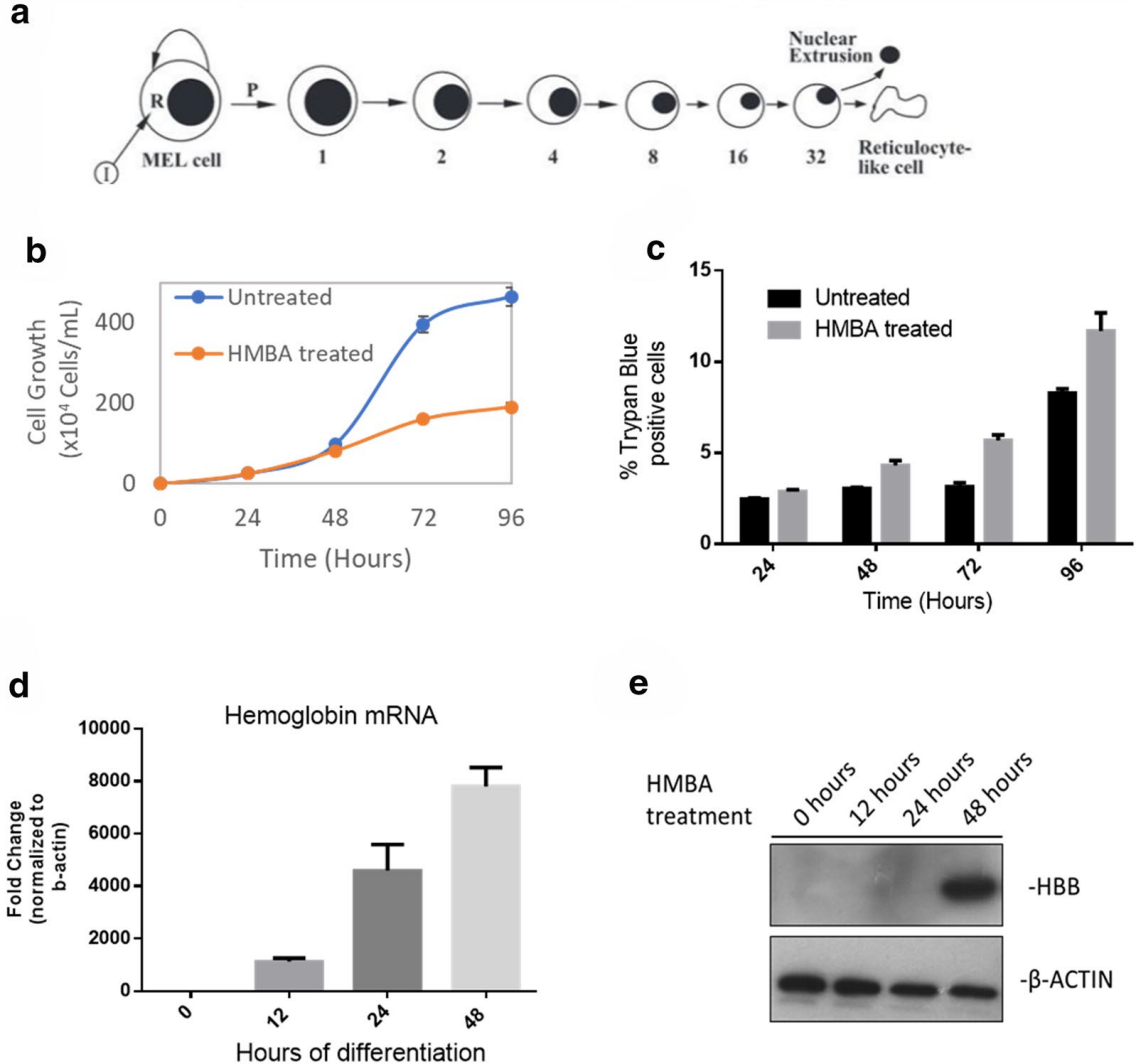
Assessment of the proliferation and differentiation capacity of MEL cells upon the induction of erythroid maturation in vitro. **a** Schematic overview of the MEL cell differentiation program to erythroid maturation in vitro (modified from [17]). Upon exposure to chemical inducers (I), the cells initiate their differentiation program to erythroid maturation with a probability (P) through the activation of crucial intracellular molecules (R). For all experiments described in this study, HMBA was used as the chemical inducer of differentiation. Committed cells lose their proliferative potential and may only divide 4–5 times, while they decrease in size and enucleate, under certain conditions during the final stage of differentiation, resembling to erythrocytes. **b** Proliferation of MEL cells in culture treated with HMBA in comparison to untreated MEL. **c** Cell death in untreated vs treated with HMBA cells for the indicated time points, scored by trypan blue exclusion assay. **d** Cells were induced to differentiate by treatment with HMBA and total RNA was isolated. qPCR analysis of the β-globin mRNA at the indicated time-points reveals efficient induction of differentiation. **e** Same experiment with D, although, here, total protein material was isolated and followed by Western blot analysis of the β-globin protein at the indicated time points

The erythroleukemic MEL and K562 cells are used in this study as a model of erythroid differentiation. Under normal culture conditions those cells are “locked’’ erythroid progenitors, resembling colony-forming unit (CFU-E) cells, characterized by quick proliferation rates (doubling time is approximately 14–18 h) and no expression of hemoglobin. Upon induction with a small molecule, such as hexamethylene bis-acetamide (HMBA), cells can re-initiate their differentiation program by limiting their proliferation potential and gradually transforming into erythrocyte-like cells (Fig. 1a, b). To better characterize our model, we treated MEL cells with 5 × 10^− 3^ M HMBA and profiled the expression of the best erythroid marker, β-globin, at the mRNA as well as at the protein level. We showed that, by 48 h of treatment, cells already produce vast amounts of hemoglobin, indicative of erythroid differentiation (Fig. 1d, e). Finally, as shown previously, differentiation is accompanied by mild induction of cell death (Fig. 1c). Thus, our model represents an appropriate tool to study the erythroid related effects of the epigenetic inhibitors.

### Assessment of the cellular proliferation capacity of MEL cells treated with the protein methyltransferase inhibitors in culture

**Fig. 2:**
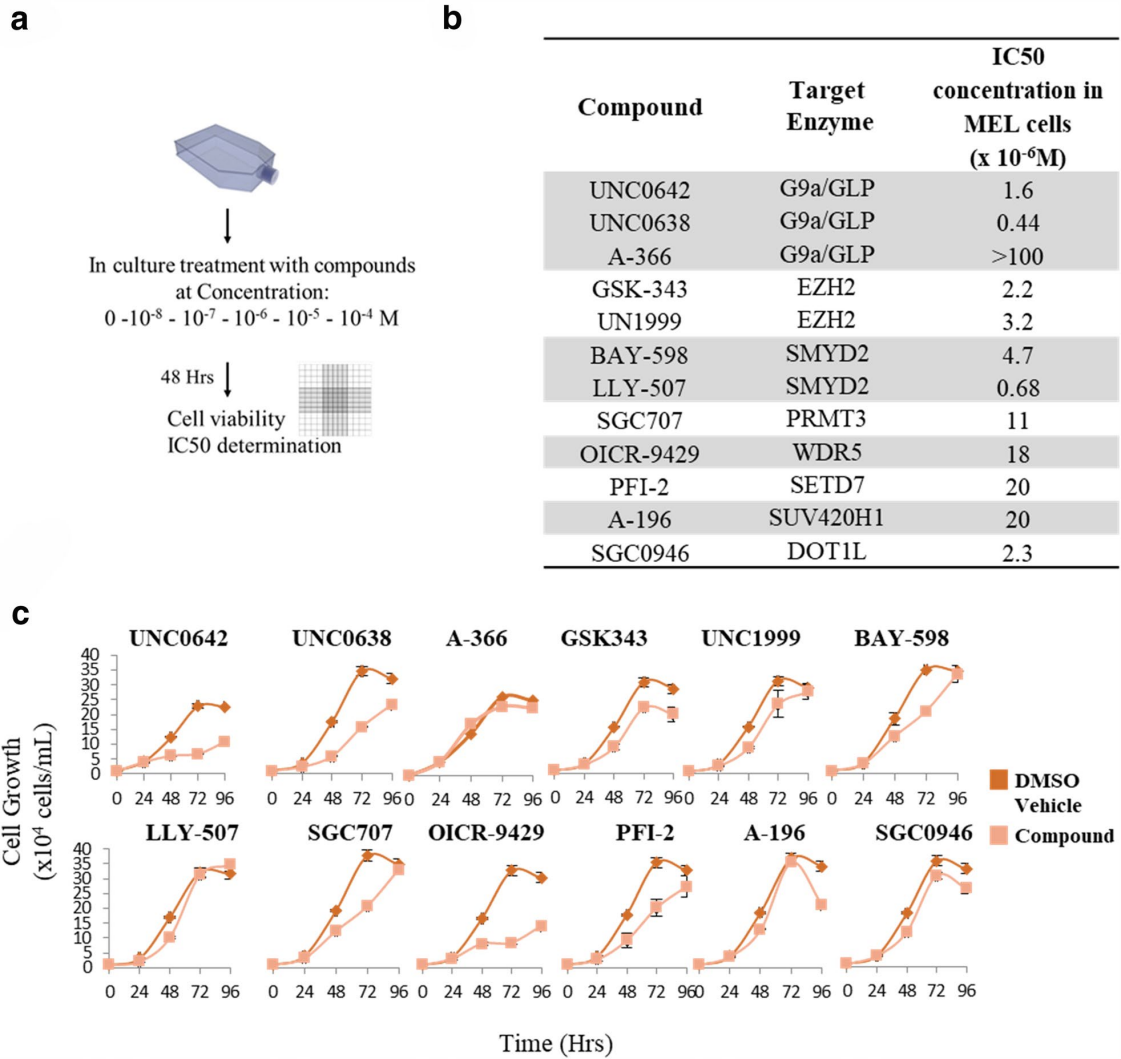
Assessment of the effects of the epigenetic probes on MEL cell proliferation in culture. **a** Depiction of the protocol followed for the determination of the IC_50_ concentration of the probes. **b** Table showing each compound’s target as well as the identified IC_50_ concentration. **c** Time-dependent kinetic analysis of the effect of the 12 compounds on the ability of MEL cells to proliferate in culture. Cells were treated continuously with each of the compounds shown in the individual diagrams at their corresponding IC_50_ concentration for each probe, and the total cell number was determined daily by cell counting via a Neubauer chamber. All but A-366 compounds showed a mild to severe inhibition of cell proliferation in culture

 In order to screen the activity of the various epigenetic probes (Table 1) in MEL cells, we carried out a concentration-dependent pharmacological assay aiming to evaluate their IC_50_ (concentration at which the 50% of growth is observed) values. For this purpose, we treated cell cultures separately with each compound in the following concentrations: 10^− 8^ M, 10^− 7^ M, 10^− 6^ M, 10^− 5^ M and 10^− 4^ M and determined cellular proliferation by cell counting 48 h after the initiation of the experiment (Fig. 2a). The ability to inhibit proliferation varied between the 12 compounds, with UNC0638 being the most toxic compound (IC_50_ = 4.4 × 10^− 7^ M), while PFI-2 and A-196 showed limited inhibitory capacity (IC_50_ = 200 × 10^− 7^ M, Fig. 2b). Interestingly, A-366 showed no effect on cellular growth and therefore the IC_50_ for this compound could not be determined (it is considered > 10^− 4^ M).

**Table 1:**
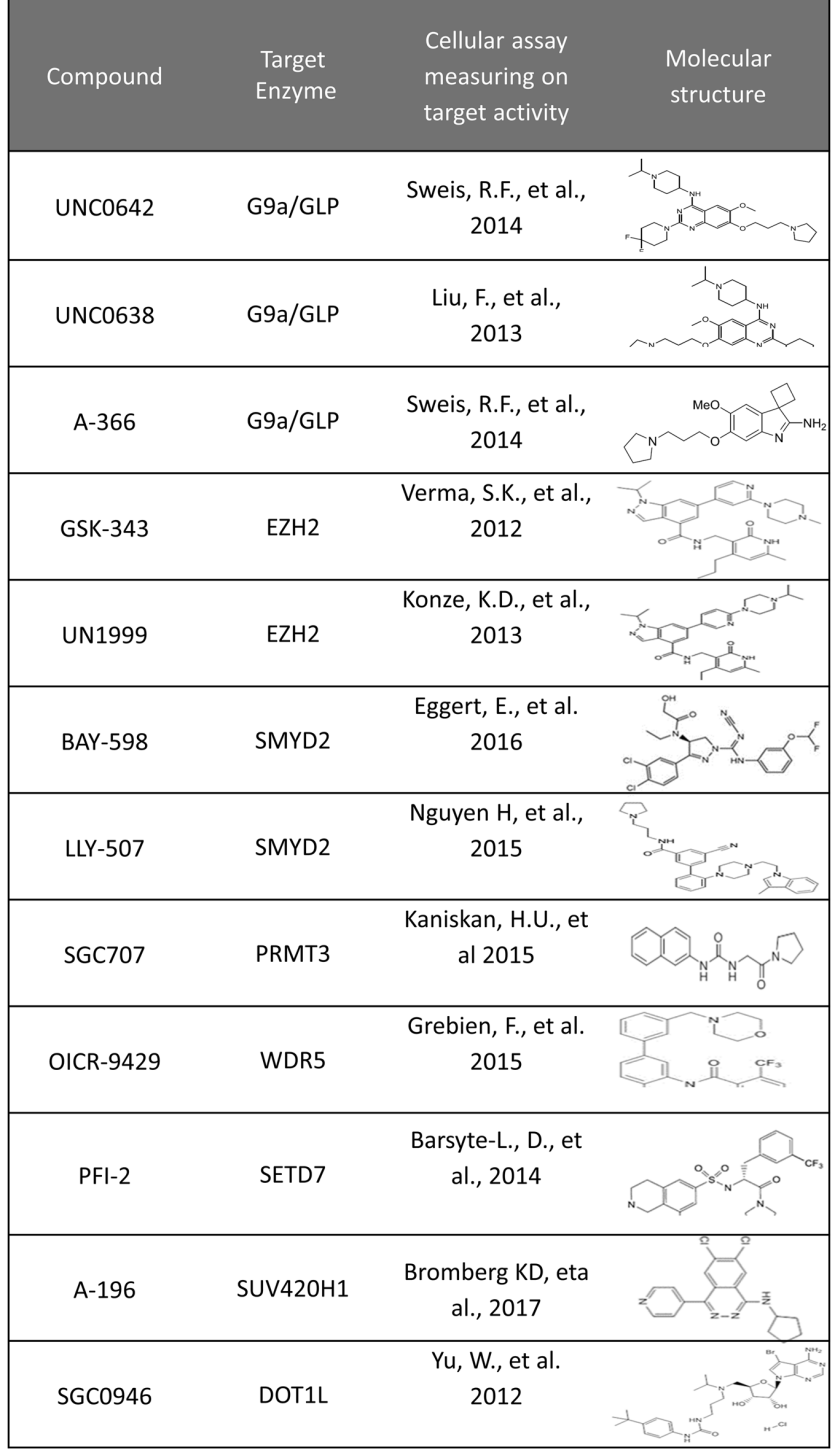
Overview of the epigenetic probes used in the study

*n* is the sum of the other counts

We, then, treated MEL cell cultures with each probe at the calculated IC_50_ concentration, at a time-dependent manner for 96 h, while monitoring daily the cell proliferation rate along with the proportion of differentiated cells accumulated in culture. Again, the effect on cellular proliferation varied, with OICR-9429 demonstrating the most cytotoxic impact of all epigenetic probes (Fig. 2c). Consistent with the IC_50_ values obtained, A-366 treatment showed no effect on the proliferation of MEL cells even after 96 h of exposure. Further, A-366 displays a significantly dissimilar pharmacodynamic profile with UNC0638 and UNC0642 despite that all compounds target the same enzyme (Fig. 2c). This data indicates that A-366 exhibits no apparent toxicity in the cell line models tested. Additionally, during the course of this experiment, no induction of differentiation has been observed in these cultures for any of the probes used, as assessed cytochemically by evaluating the number of benzidine^+^ positive cells (benzidine-H_2_O_2_ stains differentiated cells by binding to hemoglobin chains - data not shown).

### Assessment of the effects of epigenetic probes on the erythroid differentiation program of MEL cells induced by HMBA

**Fig. 3:**
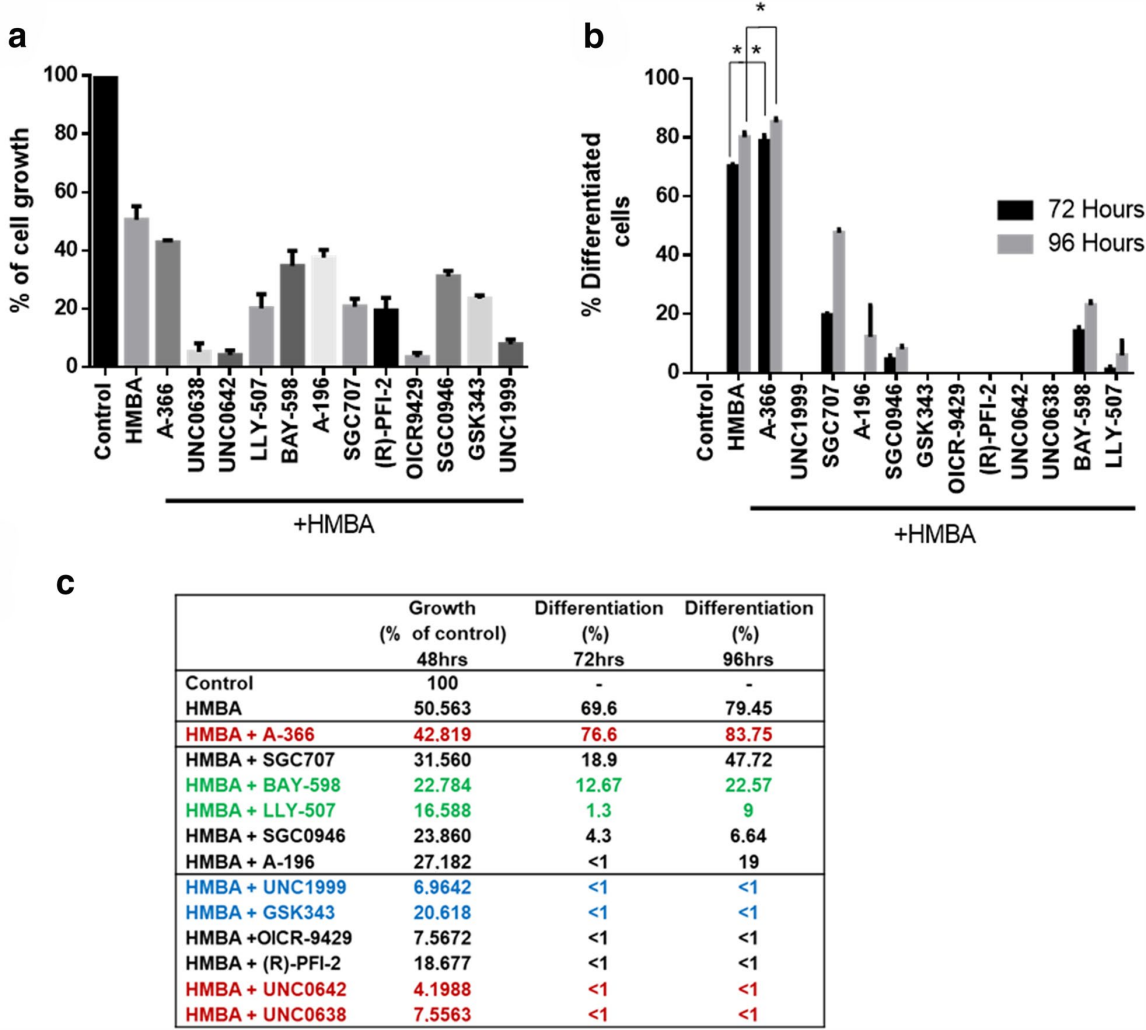
Assessment of the effect of the 12 methyltransferase inhibitors on the MEL cell erythroid differentiation program. **a** MEL cell growth determined after 48 h of treatment with the indicated compounds. For this experiment, all probes were co-administered with the inducer of differentiation, HMBA (5 × 10^− 3^ M; 0.5 mM) and cell growth was determined by cell counting (in a Neubauer chamber). **b** MEL cell differentiation determined after 48 h and/or 72 h of treatment with the indicated compounds at a concentration equal to their IC_50_ (shown in Fig. 2b). For this experiment, all epigenetic probes were co-administered with the inducer of differentiation, HMBA. Cell differentiation was determined by staining with benzidine and scoring benzidine-positive cells in culture. At least 250 cells were scored as positive or negative in each technical replication, and three technical replications were performed for each sample. **p < 0.005; *p < 0.01; Student’s t-test. **c** Table summarizing the information of **a** and **b**. Each experiment was conducted in at least three biological replications. Data in all panels represent means ± standard deviation

As stated previously, MEL cells can be induced to differentiate in culture after treatment with HMBA and show clear signs of erythroid maturation (decrease in size, limited proliferation capacity, hemoglobin expression) already by 48 h of treatment. To determine the impact of the compounds on erythroid differentiation, we co-treated MEL cells with each compound along with HMBA and determined cellular proliferation and differentiation at various times points after initiation of the experiment. As expected, HMBA treatment alone effectively induced erythroid differentiation (Fig. 3b) at approximately 80% of cells after 96 h of treatment and limited proliferation rate at approximately 50% compared to untreated cells (Fig. 3a). As for the molecular probes, the UNC0642-HMBA co-administration was the most cytotoxic compound combination, while A-366-HMBA combination merely impacted cell proliferation, compared to HMBA alone (Fig. 3a). Notably, we observed that despite most probes demonstrated an inhibitory effect on cell differentiation, A-366 facilitated the differentiation process, as measured by the cytochemical assessment of the differentiated cells by the benzidine assay (measurement of hemoglobin production) (Fig. 3b). Indeed, the co-administration of A-366 with HMBA was more effective in inducing differentiation compared to HMBA alone, both after 72 and 96 h of treatment (Fig. 3c).

**Fig. 4:**
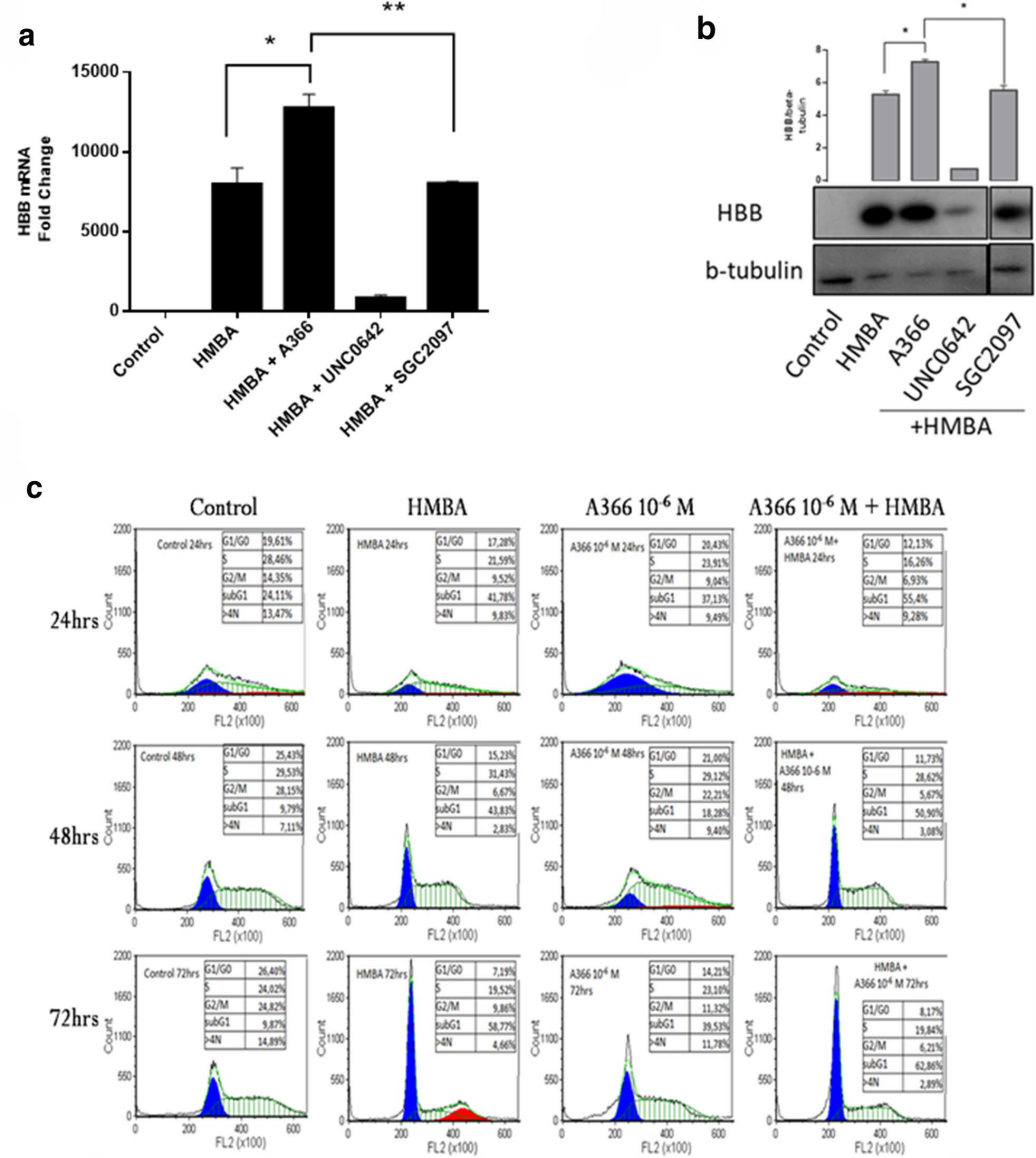
A-366 alters the cell cycle state of growing and differentiating MEL erythroid cells. Agent concentration for all panels, unless indicated differently: HMBA (5 × 10^− 3^ M), A-366 and SGC2097 (10^− 6^ M), UNC0642 (1.6 × 10^− 6^ M). **a** MEL cells were treated for 48 h with drug combinations indicated in the graph. Total RNA isolation was performed, and RNA was processed with cDNA synthesis and qPCR analysis of the β-globin transcripts. Data was normalized to β-actin expression and analyzed according to the 2^−ΔΔCt^ method. **p < 0.001; *p < 0.01; Student’s t-test. **b** MEL cells were treated for 48 h with the indicated drug combinations in the graph, before total protein isolation was performed. Western blot with specific antibodies against β-globin and β-tubulin is shown. *p < 0.05; Student’s t‐test. **c** Flow cytometry analysis of the indicated in the graph drug combinations for 24 h, 48 h and/or 72 h. Graph shows the distribution of single cells (MEL cells) into the phases of the cell cycle. Data in all panels represent means ± standard deviation

To validate the data obtained, we repeated the same co-administration scheme of A-366 with HMBA in MEL cultures and then isolated both total cytoplasmic RNA and proteins from the treated cells. Interestingly, we observed that, consistent with the colorimetric benzidine assay, both qPCR as well as western blot analysis confirmed that the A-366-treated MEL cells accumulate higher amounts of hemoglobin mRNA (β-major) and protein, respectively (Fig. 4a, b). In order to validate that this property of A-366 relies on an on-target effect, we carried out the same experiment by using the negative probe SGC2097 (negative control of A-366 that does not bind to the intended target) at the same concentration (10^− 6^ M). Co-treatment of HMBA with SGC2097 did not increase hemoglobin mRNA (β-major) and protein levels in MEL cells, as indicated by qPCR and western blot analysis (Fig. 4a, b). This effect of SGC2097 suggests that the A-366 mediated phenomena in the MEL erythroid maturation program are due to on-target activity of the compound. Taken together, A-366 demonstrates a pro-differentiation role in MEL cells, together with a lack of apparent toxicity even at high compound concentrations.

### A-366 modulates the cell cycle state of MEL cells

As it has been previously shown, the initiation of erythroid differentiation is associated with a cellular exit from the cell cycle [17, 31–33]. To test whether A-366 impacts on the cell cycle, we performed flow cytometry analysis by staining cells with propidium iodide [34]. As expected, HMBA treatment induced exit from the cell cycle, as demonstrated by a reduction in the number of cells that accumulate at the S and G2/M phases, in comparison to the control-untreated cultures (Fig. 4c). Furthermore, previous studies have shown that differentiation is accompanied by a significant increase in cells residing at the subG1 state of the cell cycle, a phenomenon which likely represents a result of chromatin condensation during erythroid differentiation [32, 35, 36]. This feature is present in the cultures treated with HMBA and increases in a time- dependent manner, as subG1 cells increase from 41.78% at 24 h treatment to 58.77% at 72 h of HMBA treatment (Fig. 4c). Interestingly, A-366 seems to mimic this behavior, by increasing subG1 cells, when administered alone compared to control-untreated cells, and also when co-administered with HMBA compared to HMBA alone cultures (Fig. 4c). This data suggests that A-366 mimics the initial HMBA effects; although not inducing differentiate if administered alone, it appears to prime cells to differentiate. Taken together, the flow cytometric data is complementing the results obtained by benzidine assay, qPCR as well as western blot, by further supporting the finding that A-366 administration facilitates erythroid differentiation of MEL cells. Moreover, the highly interesting effect of A-366 on the cell cycle suggests that the compound may function by modulating the ability of the cells to progress through the phases of the cell cycle.

### A-366 treatment modulates the expression of key cell cycle regulators

To obtain further insights into the association between A-366 and the cell cycle state of erythroid cells, we analyzed whether A-366 influences the expression of a panel of key cell cycle regulators. Previous research has shown that the activity and the levels of cyclin dependent kinases as well as the p53/p21 axis are strictly regulated during MEL and K562 differentiation [31, 32, 37–41]. Moreover, downregulation of CDK6 is regarded as an early event that is necessary for erythroid cell differentiation [31]. Thus, we monitored the expression of selected cell cycle and apoptosis regulators by performing real-time PCR (qPCR) analysis after treatment with A-366 for 48 h. The analysis was performed in the human K562 cells in order to obtain more relevant data on the physiological effects of A-366. It is also interesting to note that the highest G9a protein expression levels was found in K562 cells among a number of various cancer cell lines assessed. As a matter of fact, a recent pharmacological analysis testing newly developed G9a inhibitors was carried out in K-562 cells, in which the inhibitor DCG066 was found to induce apoptosis through blocking cell cycling at the phase G2/M [42].

**Fig. 5:**
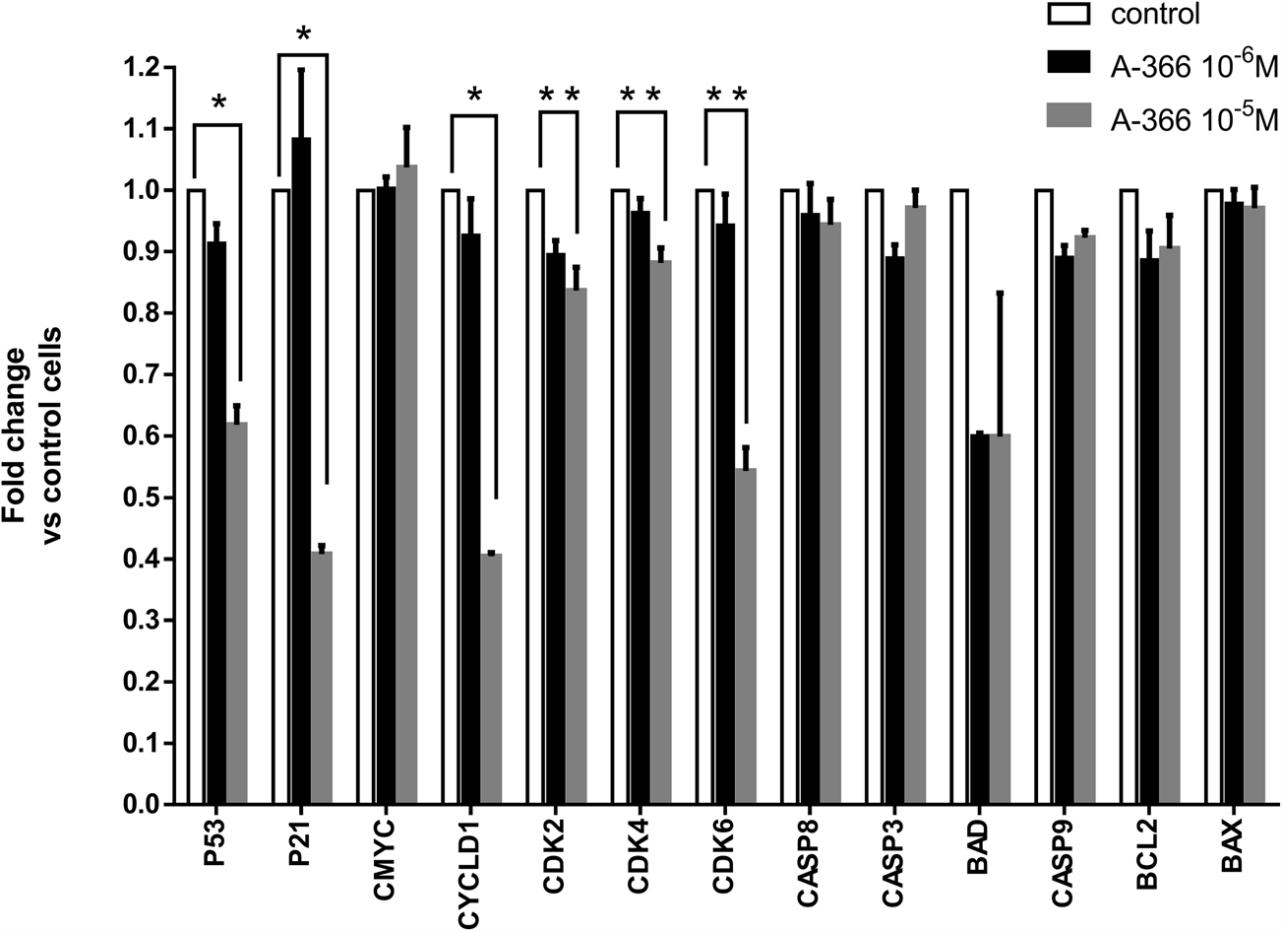
Gene expression assessment by qPCR analysis of K562 cells treated with A-366. K-562 cells (10^5^ cells ml^− 1^) were grown in the absence (control) of presence of A-366 at the concentration of 10^− 6^ M or 10^− 5^ M for 48 h. Total RNA isolation was followed by cDNA synthesis to allow qPCR analysis of the indicated genes, as shown under “Methods” section. *p < 0.01, **p < 0.05; Student’s t-test. Data in the panel represent means ± standard deviation

The qPCR analysis revealed a significant downregulation of the p53/p21 axis in A-366 treated cells (Fig. 5). This is indicative of a pro-differentiation role of A-366, as it has been previously shown that an early downregulation of the p53 pathway is a prerequisite for erythroid differentiation [37]. Moreover, we found that A-366 decreases the mRNA levels of cyclin D1, CDK2, CDK4 and CDK6 to a variable degree ranging from 10 to 60% downregulation (Fig. 5). Notably, CDK6 was significantly downregulated (approximately at 50% of the initial levels) consistent with previous studies connecting CDK6 reduction with differentiation (Fig. 5) [31, 32, 39, 40]. On the contrary, A-366 merely impacts the effectors of apoptosis, as the expression levels of caspase 3, caspase 8, caspase 9, Bcl-2 and Bax remain almost unchanged (Fig. 5). This is consistent with the hypothesis that A-366 induces only the early alterations required for differentiation, given that while apoptosis is activated during differentiation this happens only at the late stages of the process. Taken together, this data enhances the notion that A-366 manifests a modulatory role in the cell cycle state.

### Bioinformatic analysis of Spindlin1 target genes

**Fig. 6:**
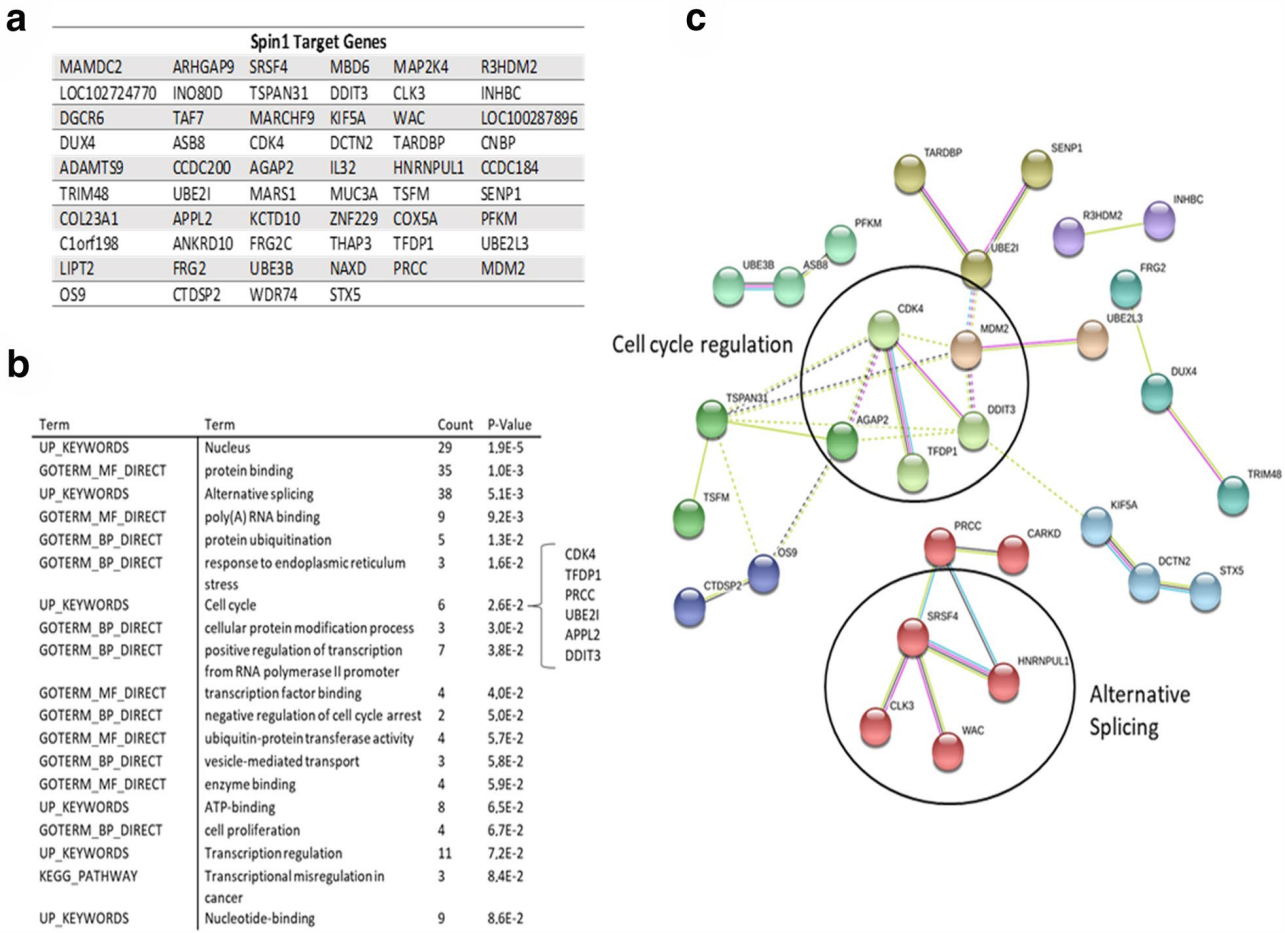
Bioinformatic analysis of the Spindlin1 target genes. **a** List depicting the predicted genes bound by Spindlin1 in human cells, retrieved from the ChIP-Atlas database. **b** Functional annotation analysis of the identified in genes shown in **a**. The analysis was performed using the DAVID bioinformatics software. **c** Protein–protein interaction network of the Spindlin1 target genes. Analysis was performed using the string database

We, finally, tried to identify additional genetic mediators of the A-366 function. According to our data, A-366 manifests a distinctive pharmacodynamic profile compared to the other available G9a inhibitors (UNC0642 and UNC0638). Thus, we hypothesized that, besides G9a, A-366 may inhibit additional targets in the cell. Indeed, a publication by Wagner et al. showed that A-366 binds with high specificity to Spindlin1, a chromatin epigenetic “reader” that recognizes and binds to histone H3 trimethylated at ‘lysine-4’, as well as asymmetrically dimethylated at ‘arginine-8’ (H3K4me3 and H3R8me2a, respectively) [43]. Spindlin1 is a known activator of the Wnt signaling pathway, hence bearing tumor-promoting properties [44]. Thus, in order to further investigate the potential involvement of Spindlin1, we retrieved publicly available data from the ChIP-Atlas database, concerning the genes bound by Spindlin1 in the nuclei of human cells [25, 45]. The analysis returned 58 target genes which potentially associate with Spindlin1 (Fig. 6a). Functional annotation using the DAVID bioinformatic database [26], shows that many genes cluster together into specific functional groups. The most populated groups are characterized by nuclear localization (29 genes), protein binding capacities (35 genes), as well as modulation of alternative splicing (38 genes). Furthermore, we identified six genes (PRCC, UBE2I, CDK4, APPL2, DDIT3 and TFDP1) with a cell cycle modulatory role (Fig. 6b). Of note, the CDK4 gene (which was found downregulated in A-366 cells) belongs to the Spindlin1 target group, thus validating our in vitro findings. Finally, we constructed a protein-protein interaction network to test whether the Spindlin1-regulated genes may functionally cooperate within the cell. Indeed, we found that two clusters of proteins appear, the first characterized by cell cycle modulatory ability, whereas the second affects alternative splicing (Fig. 6c). Taken together, A-366 seems to operate by inhibiting Spindlin1 and therefore potentially affecting the group (or a sub-group) of the identified 58 genes. The analysis performed indicates that the effects of A-366 are mediated by (a) altering the expression of critical cell cycle regulators (such as CDK2, CDK4, CDK6, UBE2I and others) and (b) affecting gene expression via alternative splicing.

## Discussion

Epigenetic alterations, in the form of DNA methylation, histone methylation and acetylation, contribute to the execution of complex cellular procedures in health and disease [6, 17, 19, 20, 45]. Moreover, epigenetic dysregulation has been recognized as one of the hallmarks of cancer cells, and a growing body of research has linked the actions of various types of epigenetic enzymes with both cancer initiation and progression [46]. The generation of chemical probes able to specifically bind and inhibit epigenetic enzymes promises to deliver novel therapeutic solutions in cancer treatment [47–49]. Therefore, there is growing interest in characterizing the effects of the generated chemical probes in relevant cell and animal models.

The work in this study points to the very interesting properties of A-366, a probe known to target G9a which was recently revealed to, also, bind Spindlin1. The results indicate that A-366 had no impact on the cell growth of erythroleukemia MEL and K-562 cell lines, while UNC0638 and UNC0642 (which also target G9a) exhibited anti-proliferative effects. The same result for A-366 was also observed in an unbiased screen of 38 cell lines, including K562, originating from numerous cancer types [50]. According to this screening, A-366 had no overt antiproliferative effects in any cell line tested up to 10 µM, often in contradiction to the inhibitor UNC0638 [50]. Moreover, A-366 demonstrated no toxicity to a mouse xenograft model when administered via osmotic mini-pump at 30 mg kg^− 1^ day^− 1^ for 2 weeks [50]. Thus, A-366 is a non-toxic compound as shown here and in an independent study.

The erythroid differentiation program is characterized by cell cycle exit and accumulation of cells into a dormant G0 state, accompanied by extensive chromatin condensation [1, 17, 19]. The contribution of the three main cyclin-dependent kinases, CDK2, CDK4 and CDK6, has been extensively studied in MEL and K562 cell erythroid differentiation programs. CDK6 activity and levels are downregulated during the early stages of differentiation, while CDK2 and CDK4 levels decline during the final stages [31, 39, 40]. In our experiments, we observed a significant A-366-dependent downregulation of CDK6, and a moderate decrease of CDK2 and CDK4. Thus, the data favors the possibility that A-366 facilitates cell differentiation by promoting some of the early events (such as the CDK6 downregulation) required for erythroid differentiation. Moreover, although, A-366 may moderately alter the expression of some of the effectors of the late stages, such as the CDK2 and CDK4, this does not seem to be coupled with activation of additional key events during late erythropoiesis, such as apoptosis, as shown by qPCR analysis of the apoptosis involved genes. In conclusion, this data suggests that A-366 contributes to the early events required for initiation of commitment to erythroid maturation.

Moreover, the contradictory behavior between A-366 and UNC0642–UNC0638 in our experiments suggests that the A-366 effects in MEL differentiation is likely a result of Spindlin1 inhibition. Accordingly, Pappano et al. found that only A-366 had a differentiation inducing role in promyelocytic cells, despite also testing UNC0638 [50]. Furthermore, our data indicates that the A-366 effects are a product of altered expression of several cell cycle regulators, including CDK4 which is, also, a Spindlin1 target. A more detailed analysis of the nuclear Spindlin1 targetome revealed an ability to bind to two main networks of genes: (a) six genes involved in cell cycle regulation and (b) numerous genes implicated with alternative splicing. Therefore, A-366 may affect a network of 58 genes, with cell cycle and splicing modulating properties and, hence exert a differentiation promoting function in erythroid as well as promyeloid cells. The recent research efforts to synthesize additional specific spindlin1 inhibitors could permit to further test and validate this hypothesis.

## Conclusions

The work presented in this study, provide new insights on the compelling effects of A-366 in cellular erythroid differentiation models. A-366 has no apparent toxicity even when administered at high concentrations, in contrast to a group of additional highly specific epigenetic probes tested. More significantly, it acts as a pro-differentiation agent by manipulating the cell cycle and inducing some of the early alterations required for erythroid differentiation. Taken together, the properties of A-366 hold promise for further potential pharmacological and clinical exploitation of the compound in erythroleukemia.

## Data Availability

Please contact the principal investigator for data requests.
